# Machine Learning Algorithms Applied to Semi-Quantitative Data of the Volatilome of Citrus and Other Nectar Honeys with the Use of HS-SPME/GC–MS Analysis, Lead to a New Index of Geographical Origin Authentication

**DOI:** 10.3390/foods12030509

**Published:** 2023-01-22

**Authors:** Ioannis Konstantinos Karabagias, Gulzar Ahmad Nayik

**Affiliations:** 1 Department of Food Science & Technology, School of Agricultural Sciences, University of Patras, G. Seferi 2, 30100 Agrinio, Greece; 2 Department of Food Science & Technology, Government Degree College Shopian, Jammu & Kashmir 192303, India

**Keywords:** citrus honey, volatiles, characterization, machine learning, discrimination, new index

## Abstract

The scope of the current study was to monitor if semi-quantitative data of volatile compounds (volatilome) of citrus honey (ch) produced in different countries could potentially lead to a new index of citrus honey authentication using specific ratios of the identified volatile compounds in combination with machine learning algorithms. In this context, the semi-quantitative data of the volatilome of 38 citrus honey samples from Egypt, Morocco, Greece, and Spain (determined by headspace solid phase microextraction coupled to gas chromatography mass spectrometry (HS-SPME/GC–MS)) was subjected to supervised and unsupervised chemometrics. Results showed that honey samples could be classified according to the geographical origin based on specific volatile compounds. Data were further evaluated with additional nectar honey samples introduced in the multivariate statistical analysis model and the classification results were not affected. Specific volatile compounds contributed to the discrimination of citrus honey in different amounts according to geographical origin. These were lilac aldehyde D, dill ether, 2-methylbutanal, heptane, benzaldehyde, α,4-dimethyl-3-cyclohexene-1-acetaldehyde, and herboxide (isomer II). The numerical data of these volatile compounds was summed up and divided by the total semi-quantitative volatile content (R_ch_, Karabagias–Nayik index) of citrus honey, according to geographical origin. Egyptian citrus honey had a value of R_ch_ = 0.35, Moroccan citrus honey had a value of R_ch_ = 0.29, Greek citrus honey had a value of R_ch_ = 0.04, and Spanish citrus honey had a value of R_ch_ = 0.27, leading to a new hypothesis and a complementary index for the control of citrus honey authentication.

## 1. Introduction

A major concern of the food sector and research community nowadays is to find the most appropriate strategy to authenticate foods. The term authentication is rather complicated. It involves the botanical and geographical origin determination of foods using specific compositional criteria and properties [1]. Products with a specific composition and origin demand a higher price in the international market. For example, there are over 6500 scientific papers dealing with honey worldwide [2,3,4,5,6]. 

*Apis mellifera* honeybees produce citrus honey when beekeepers place the beehives in citrus groves during the blossom period. The citrus nectariferous species involved dynamically in the production of citrus honey are oranges (*Citrus sinensis* L.), followed by bitter orange (*Citrus bigaradia* L.), tangerines (*Citrus tangerina* or *Citrus reticulata*), and lemons (*Citrus limon* L.). This type of honey has some special characteristics. These include its intense aroma, mild flavor, light amber color, and quick crystallization. Some statistics about the production rate show that this type of honey accounts for 10% of the annual honey production in Greece. Important amounts of citrus honey are also produced in Italy, Spain, Mexico, France, the USA, Israel, Egypt, and Morocco [5]. The percentage of pollen grains in citrus honey is often under-represented, and therefore is practically impossible to use only pollen data as a measure of the typification of citrus honey. This is supported by a wide range of pollen grain percentage reported for European and Mediterranean citrus honey (ca. 2–42%) [2,5,6]. In this context, physicochemical parameter analysis (electrical conductivity, pH, ash, acidity, minerals, sugars, volatile compounds, flavonoids, etc.), especially in the form of “physicochemical markers”, identified using multivariate statistics, sensory analysis, or the correlation of citrus pollen grains with volatile compounds, are adopted by the research community to deeper characterize this type of honey [3,4,5,6,7,8,9,10,11]. 

The solid-phase microextraction (SPME) technique was first introduced by Pawliszyn [12]. Until then, numerous studies were carried out on foods regarding determination of volatile compounds, including honey, which is rather a complicated matrix. The technical nature of SPME is that it is a sample preparation technique, which combines the usage of a fused-silica fiber that is coated on the outside with an appropriate stationary phase. The analyte in the sample is directly extracted to the coating of the fiber. The SPME technique can be applied, in fact routinely, in combination with other analytical techniques such as gas chromatography (GC), gas chromatography–mass spectrometry (GC–MS), high-performance liquid chromatography (HPLC), or high-performance liquid chromatography coupled to mass spectrometry (LC–MS) [13]. SPME can be recognized as a “green” technique that, in combination with analytical chemical techniques, can rapidly provide adequate data regarding the volatile pattern of foods. The volatile markers of citrus honey that have been associated previously with its typicity include methyl anthranilate, isomers of lilac aldehydes, hotrienol, α,4-dimethyl-3-cyclohexen-1-acetaldehyde, dill ether, nerolidol, 1-para-menthen-9-al, linalool, DL-limonene, (E)-limonene oxide, ethyl linalool, etc. [3,4,5,6,7]. 

Taking the aforementioned into consideration, the scope of the current study was to characterize and discriminate citrus honey from different countries using semi-quantitative data of volatile compounds in combination with machine learning algorithms. The obtained multivariate discriminating models were further evaluated using a set of honey samples containing other nectar honey types such as flower and thyme honey. Finally, an effort was made to establish a new metric for citrus honey authentication using the selected volatile compounds from the multivariate analysis and producing specific ratios. In the recent literature, this is the first attempt to establish such a metric index for citrus honey authentication, comprising the originality of the present study and providing support to previous studies in the relevant literature.

## 2. Materials and Methods

### 2.1. Honey Samples

Thirty-eight citrus honey samples (*Citrus* spp.) as confirmed by melissopalynological analysis [2,14] (Supplementary Material, Section I), were subjected to HS-SPME/GC–MS analysis. The honey samples were harvested in the year 2013–2014 and collected from Egypt, Morocco, Greece, and Spain. Honey samples from Egypt, Morocco, and Spain were purchased from local markets and shipped to the laboratory. Honey samples from Greece were donated by Attiki Bee Culturing Co. Melissopalynological analysis showed that honey samples had a diverse level of pollen grains of the citrus species ranging between 8 and 32% with respect to citrus honey geographical origin. For validation of the statistical analysis models used for the determination of geographical origin of citrus honey, six additional honey samples from the same harvesting year donated by professional beekeepers from Aitoloakarnania (2 flower honey samples) and Kefalonia (4 thyme honey samples) were introduced into the analysis. These samples were grouped as “nectar honey” samples. The electrical conductivity of flower honey samples was 0.28 ± 0.01 mS/cm and that of thyme honey samples 0.45 ± 0.02 mS/cm, in accordance with the values indicated by the council directive relating to nectar honey [1]. All samples were stored in glass and dark metal containers protected from light at room temperature (20 ± 1 °C) and analyzed soon after the collection.

### 2.2. Reagents and Solutions

Absolute ethanol (CH_3_CH_2_OH) and sodium chloride (NaCl) were purchased from Merck (Darmstadt, Germany). Benzophenone [(C_6_H_5_)_2_CO, molecular weight = 182.22] was purchased from Sigma-Aldrich (Germany). The standard mixture of *n*-alkane solution (C8-C20), dissolved in *n*-hexane, used for the determination of Kováts indices (experimental indices related to the retention time of each volatile compound) was purchased from Supelco (Bellefonte, PA, USA).

### 2.3. Determination of Volatile Compounds

Volatile compounds of citrus and nectar honey samples were determined using HS-SPME/GC–MS following a “green” procedure, since only distilled water was used for the dilution of honey samples and the extraction of volatile compounds. 

#### 2.3.1. Extraction of Volatile Compounds 

The extraction of headspace volatile compounds of citrus and nectar honey samples was carried out with the use of a divinyl benzene/carboxen/polydimethylsiloxane (DVB/CAR/PDMS) fiber (50/30 μm) (Supelco, Bellefonte, PA, USA). Before using the fiber for the extraction of headspace volatile compounds, conditioning was mandatory according to the manufacturer’s recommendations. The headspace extraction of volatile compounds was accomplished using an optimized protocol [15]. For the preparation of samples, 2 g of honey was dissolved in 2 mL of distilled water, followed by the addition of 0.20 g sodium chloride (NaCl, Merck, Darmstadt, Germany) and 20 μL of benzophenone as internal standard (100 μg/mL) (Sigma-Aldrich). Samples were placed in vials of 15 mL volume, with a screw-cap and equipped with septa of PTFE/silicone. A magnetic stir bar (cross-shaped PTFE-coated magnetic stir bar of 10 mm diameter (Semadeni, Ostermundigen, Bern, Switzerland) was also placed inside each vial to assist in the extraction of volatile compounds. Afterwards, the vials were placed in a water bath (45 °C) and stirring at 600 rpm was applied during the headspace extraction of volatile compounds. To avoid any contamination that could cause false results, the fiber was cleaned daily before use with the “clean-program” method [15], and blank runs were carried out during the consecutive analysis of honey samples. Each sample was run in duplicate (in total 88 runs).

#### 2.3.2. Instrumentation and Analytical Conditions

The GC–MS unit used for the analysis of volatile compounds of honey samples was supplied by Agilent (GC unit, Agilent 7890 A; MS detector, Agilent 5975). The capillary column used for the separation of volatile compounds was a DB-5MS (cross linked 5% PH ME siloxane) capillary column (60 m × 320 μm i.d., ×1 μm film thickness), and helium of high purity (purity 99.999%) was used as carrier gas at a flow rate of 1.5 mL/min. 

Regarding injector and MS-transfer line, these were maintained at 250 °C and 270 °C, respectively. The oven temperature was held at 40 °C for 3 min, and then increased to 260 °C at a rate of 8 °C/min (6 min hold) [15]. Electron impact mass spectra were recorded in the range of 50–550 amu. Ionization energy was 70 eV and a split ratio of 1:10 was used.

#### 2.3.3. Identification of Volatile Compounds 

Volatile compounds of honey samples were identified using the Wiley 7, NIST 2005 mass spectral library. Kováts indices were calculated for components eluting between *n*-octane and *n*-eicosane. Volatile compounds that had ≥90% similarity with the Wiley mass spectral library were identified using GC–MS spectra. Data were then expressed as semi-quantitative content in micrograms per kilogram (Canalyte, mg/kg) according to the equation:

Canalyte (mg/kg) = (Eanalyte)/(Ebenzophenone) × C_IS_ (1)

where, Eanalyte and Ebenzophenone are the peak areas of the isolated volatile compounds and internal standard, respectively. C_IS_ is the concentration of the internal standard (1 mg/kg).

Semi-quantification was carried out using benzophenone (*m*/*z* = 182) and assuming a response factor (RF) equal to one (RF = 1) for the identified volatile compounds [7]. The semi-quantitative results were expressed as average ± standard deviation (Avg ± SD). In addition, benzophenone had a good yield in its area (over 95%) among replicated samples. Kováts indices were calculated for all determined volatile compounds and values were compared with those in the literature.

### 2.4. Statistical Analysis: Machine Learning Theory

The semi-quantitative data of volatile compounds (mg/kg) were subjected to statistical analysis to investigate the impact of geographical origin on the volatile composition of honey samples. The comparison of the average values was performed using multivariate analysis of variance (MANOVA) to determine which volatile compounds showed significant differences (*p* < 0.05) in their composition among honey samples of different geographical origin (that is citrus honey from Egypt, Morocco, Greece, and Spain, and nectar honey from Greece). Regarding MANOVA, it creates a new dependent variable based on linear combination of all the dependent variables introduced in the model, which maximizes the differences in the average values between the level groups of the independent variable as far as possible. Wilks’ lambda and Pillai’s trace criteria were implemented to study the main effects and interaction of the independent variables at the multidimensional level [16]. The robustness of the sample size used in the experiment was estimated using the observed power and power analysis. Power is the probability of rejecting the general hypothesis that the means are equal, when these are in fact not equal. The power during MANOVA depends on the sample size, the magnitudes of the variances, the alpha level, and the differences among the population means in a given group of objects. In that sense, high power is desirable. High power means that there is a high probability of rejecting the null hypothesis when the null hypothesis is false. This is a critical measure of precision in the aforementioned hypothesis during the application of multivariate statistics [17]. LDA which is a supervised statistical technique, was then only applied to the significant (*p* < 0.05) volatile compounds (as a follow up test to MANOVA) to determine a linear combination of this group of subjects which could differentiate citrus/and or nectar honey correctly according to geographical origin (independent variables). For comparison and to extract, if possible, some additional/and or dynamic information, principal component analysis (PCA) was applied. PCA is an unsupervised statistical technique that is used in exploratory data analysis and for making predictive models. It is commonly used for dimensionality reduction by projecting each data point onto only the first few principal components to obtain lower-dimensional data, while retaining the variation of the data as much as possible. The first principal component can equivalently be defined as a direction that maximizes the variance of the projected data. The *i_t_*_h_ principal component can be taken as a direction orthogonal to the first *i*-1 principal component that maximizes the variance of the projected data. Usually, components that have an eigenvalue greater than 1 are considered the main factor variables [18]. Pearson’s bivariate correlations (−1 to +1) and one-sample *t*-tests were used in each case at a confidence level of *p* < 0.05. Statistical analysis was carried out using SPSS software v. 27.0 (SPSS, IBM Inc., Armonk, NY, USA).

## 3. Results

### 3.1. Volatile Compounds of Citrus and Other Nectar Honeys

**Figure 1:**
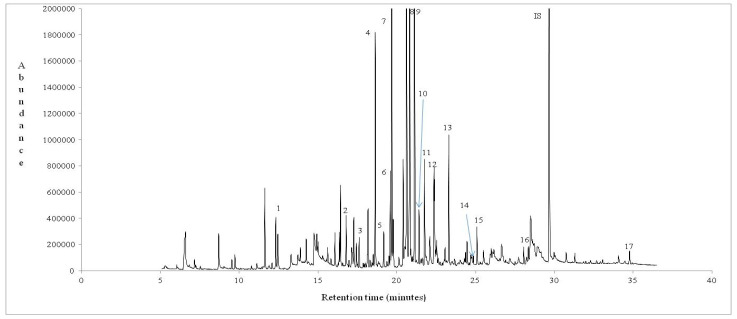
Gas chromatogram of citrus honey from Argos (Greece). Volatile compounds used as potential indicators of citrus honey geographical origin are numbered accordingly. 1: Octane. 2: Benzaldehyde. 3: Herboxide (isomer II). 4: Benzeneacetaldehyde. 5: cis-Linalool oxide. 6: Linalool. 7: Nonanal. 8: Lilac aldehyde C. 9: Lilac aldehyde D. 10: Ethyl octanoate. 11: Decanal. 12: α, 4-Dimethyl-3-cyclohexene-1-acetaldehyde. 13: Ethyl nonanoate. 14: Methyl anthranilate. 15: Ethyl decanoate. 16: Ethyl dodecanoate. 17: Ethyl hexadecanoate. IS: Internal standard.

Forty-one volatile compounds were identified in citrus and other nectar honey samples. Among these volatiles, thirty-three were identified in citrus honey samples of different geographical origin, and nine additional volatiles were identified in flower and thyme honey samples (grouped as other nectar honey samples). The volatile compounds were grouped into classes: alcohols, aldehydes, esters, ethers, hydrocarbons, ketones, phenolics, and terpenes (Table 1). The most abundant volatile compounds were aldehydes, followed by esters and the other classes of compounds. Total semi-quantitative volatile content (TSQVC) followed the order Morocco = Greece > Egypt > Spain. Figure 1 shows a representative gas chromatogram of citrus honey from Argos (Greece).

It should be stressed that of the 41 identified compounds, 30 showed significant differences between the different producing countries, also including the different honey types used for the validation of results (Table 1). This indicates that the volatile fraction of citrus or nectar honey provides potentially useful information enabling its correct geographical and botanical discrimination. In support of this finding, some volatile compounds were characteristic for the other nectar honey samples studied: 1-octanol, 1-nonanol, ethyl hexanoate, ethyl heptanoate, ethyl malonate, undecane, delta-3-carene benzeneethanol, and benzeneacetonitrile. Ethyl malonate (propanedioic acid ethyl ester), however, is for the first time reported in the literature to contribute to the aroma of flower honey from Aitoloakarnania.

### 3.2. Geographical Origin Indication of Citrus and Other Nectar Honeys Based on Volatile Compounds and Machine Learning Algorithms

#### 3.2.1. Estimation of Sample Size: Power Analysis

Prior to the statistical analysis of volatile compound data with machine learning algorithms (MANOVA, LDA, PCA), power analysis was run to check whether the sample size of honey was sufficient for the purpose of the study. The desired power was set at 0.8 (it ranges from 0 to 1). The overall test applied is based on the null hypothesis that the population mean is the same for all groups. Power analysis showed that to obtain the desired power of 0.8 (80% accuracy) the total sample size across groups should be at least n = 10. The actual power of the model was 1.000 based on the non-central F-distribution and the effect size measured by the root-mean-square standardized effect was 535.413 (*p* = 0.05). More specifically, the group size allocation for the overall test was: group 1 (Egypt) two samples; group 2 (Morocco) two samples; group 3 (Greece) three samples; and Group 4 (Spain) two samples. As it can be observed the initial sample size used in the study (n = 38) was sufficient to obtain a power/explanation of data close to 80%. 

#### 3.2.2. PCA: Characterization of Citrus Honey Samples of Different Geographical Origin (Part I)

During PCA, the volatile compounds that showed high loading values were ethyl dodecanoate (F1, loading value of 0.871), lilac aldehyde C (F2, loading value of 0.690), α-4-dimethyl-3-cyclohexene-1-acetaldehyde (F3, loading value of 0.494), lilac aldehyde D (F4, loading value of 0.657), 2-methylbutanal (F5, loading value of 0.503), methyl anthranilate (F6, loading value of 0.946), and para-cymene (F7, loading value of 0.930) (Table 2). 

**Figure 2:**
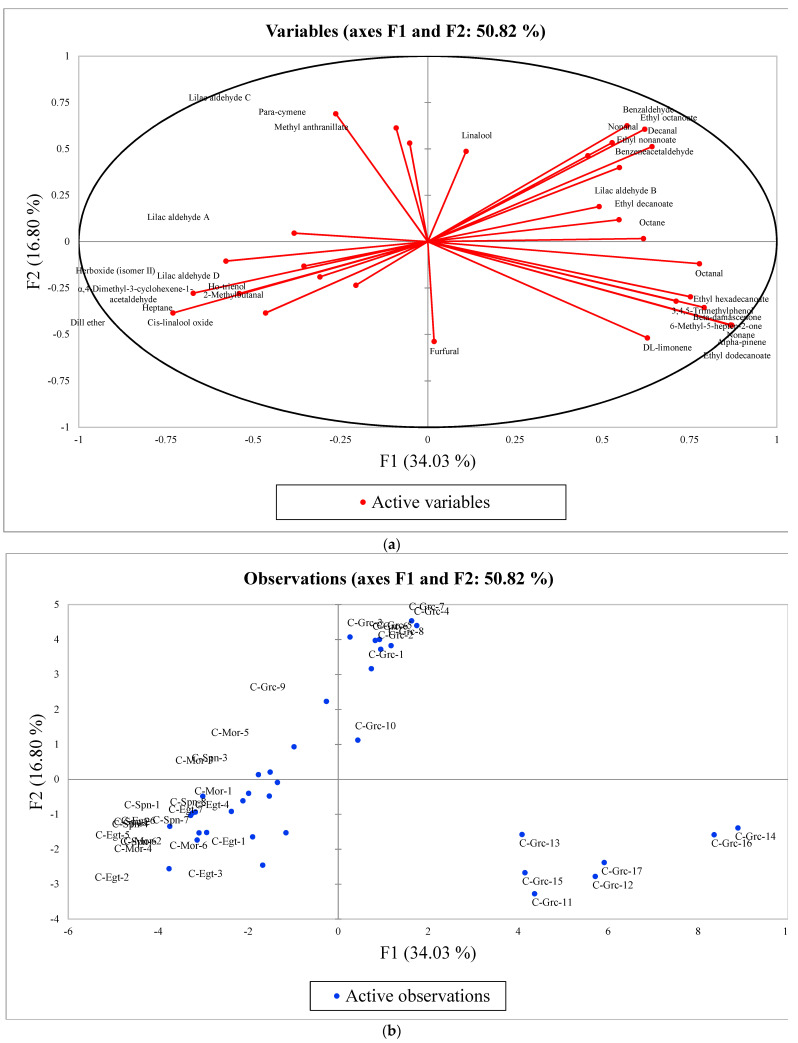
(**a**) Active volatile compounds (variables) of citrus honey related to geographical origin based on PCA. α: alpha; β: beta. (**b**) Score plot (distribution of citrus honey samples) in the multi-dimensional space of PCA with respect to geographical origin.

The first two components (factors) explained 50.82% of the total variance (Figure 2a,b). In addition, the group of samples situated at the bottom (right quadrant) indicates that citrus honey samples C-Grc-11 to C-Grc-17, belonging to the group of citrus honey from Greece, showed high positive scores for component 1, and component 1 also has high loadings for octane, α-pinene, octanal, nonane, decanal, nonanal, β-damascenone, 6-methyl-5-hepten-2-one, ethyl dodecanoate, ethyl hexadecanoate, and 3,4,5-trimethylphenol. The high loadings for volatile compounds reveal that these play a significant role in the overall variation of citrus honey from Argos (Greece) as indicators of its geographical origin. The analysis further reveals that the volatile compounds grouped together in Figure 2a,b at the bottom (right quadrant) are highly correlated.

#### 3.2.3. PCA: Characterization of Citrus and Other Nectar Honey Samples of Different Geographical Origin (Part II)

For PCA in case a higher sample size of honey was used (n = 44) (citrus and nectar honey samples) the volatile compounds that had high loading values were: α-pinene (loading value of 0.76), decanal (loading value of 0.72), benzeneacetonitrile (loading value of 0.78), lilac aldehyde C (loading value of 0.73), α,4-dimethyl-3-cyclohexene-1-acetaldehyde (loading value of 0.59), ethyl decanoate (loading value of 0.74), lilac aldehyde D (loading value of 0.62), furfural (loading value of 0.47), lilac aldehyde B (loading value of 0.54), and hotrienol (loading value of 0.64).

#### 3.2.4. LDA: Geographical Origin Discrimination of Citrus Honey (Part I)

**Figure 3:**
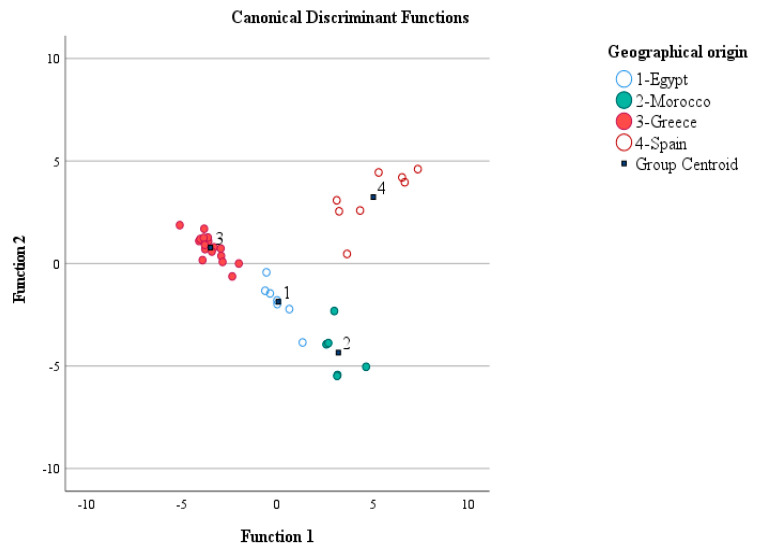
Discrimination of citrus honey geographical origin based on volatile compounds and LDA.

In this case, the geographical origin classification rates using the cross-validation method in LDA were 71.4%, 50%, 88.2%, and 87.5% for citrus honey form Egypt, Morocco, Greece, and Spain, respectively. The overall error in each case was 28.6%, 50%, 11.8%, and 12.5%. Regarding precision of the LDA classification model, from the initial seven samples from Egypt, five were allocated correctly to Egypt, one to Morocco and one to Greece. In case of citrus honey samples from Morocco, three samples were allocated correctly to Morocco, two to Egypt and one to Greece. The best classification results were obtained for citrus honey samples from Greece, since from the initial seventeen samples, fifteen were correctly allocated to Greece and two to Egypt. Finally, very good classification results were also obtained for the citrus honey samples from Spain, as from the initial eight samples seven were allocated correctly to Spain and one to Morocco (Supplementary Table S1). The discrimination of samples is shown in Figure 3. 

The volatile compounds with high discrimination power were lilac aldehyde D (absolute correlation value of 0.57), dill ether (absolute correlation value of 0.32), 2-methylbutanal (absolute correlation value of 0.41), heptane (absolute correlation value of 0.37), benzaldehyde (absolute correlation value of 0.37), α,4-dimetyhyl-3-cyclohexene-1-acetaldehyde (absolute correlation value of 0.19), and herboxide second isomer (absolute correlation value of 0.17). Full data are given in the supplementary material (Section II).

#### 3.2.5. LDA: Discrimination of Citrus and other Nectar Honeys (Part II)

For validation of the aforementioned results, and to test the initial model efficacy, MANOVA analysis was applied to the semi-quantitative data of volatile compounds of the 44 citrus and other nectar honeys again, to determine which volatile compounds were now significant concerning discrimination of their geographical origin. In this case, dependent variables included the 42 volatile compounds, since the other nectar honey samples used in the multivariate analysis had some additional characteristic volatile compounds, and the origin of honey samples (citrus honey from Egypt, citrus honey from Morocco, citrus honey from Greece, citrus honey from Spain, and other nectar honeys from Greece) was taken as the independent variable. Pillai’s trace = 3.888 (F = 6.773, df = 144, *p* < 0.001) (with observed power equal to 1.000), and Wilks’ lambda = 0.000 (F = 23.073, df = 144, *p* < 0001) (with observed power equal to 1.000) index values showed the existence of a significant multivariable effect of citrus and other nectar honeys geographical origin on their volatile composition. Thirty-one of the forty-two volatile compounds (Table 1) were significant (*p* < 0.05) for geographical origin discrimination of citrus and other nectar honeys. Afterwards, these 31 volatile compounds were subjected to LDA. LDA showed that four statistically significant discriminant functions were formed: Wilks’ lambda = 0.000, *X*^2^ = 312.976, df = 100, *p* < 0.001 for the first function; Wilks’ Lambda = 0.003, *X*^2^ = 165.143, df = 72, *p* < 0.001 for the second; Wilks’ Lambda = 0.027, *X*^2^ = 100.793, df = 46, *p* < 0.001 for the third; and Wilks’ Lambda = 0.228, *X*^2^ = 41.441, df = 22, *p* < 0.01 for the fourth.

**Figure 4:**
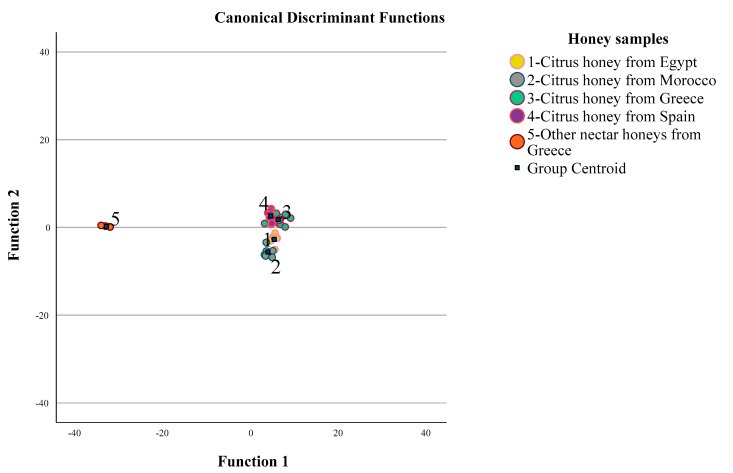
Discrimination of the geographical origin of citrus and other nectar honeys based on volatile compounds and LDA.

The first discriminant function accounted for 90.8% of the total variance and had the highest eigenvalue (195.319) and canonical correlation (0.997). The second discriminant function had a significantly lower eigenvalue (8.956) and canonical correlation (0.948), while accounting for 4.2% of the total variance. The third discriminant function had a lower eigenvalue (7.329) and canonical correlation (0.938) accounting for 3.4% of the total variance. Finally, the fourth discriminant function had the lowest eigenvalue (3.393) and canonical correlation (0.879) accounting for 1.6% of the total variance. All discriminant functions accounted for 100% of the total variance. During LDA, another important parameter is the tolerance level of the variables (volatile compounds) used for the classification of honey samples according to geographical origin. SPSS software uses a default value of 0.001 as the minimum tolerance level in LDA. The volatile compounds that had a tolerance level value of 0.000 did not pass the tolerance test, and were not included “a priori” in the discriminant functions. These volatile compounds were: ethyl malonate, 1-octanol, undecane, benzeneethanol, and benzeneacetonitrile. The group centroid values were: (5.213, −2.730), (3.774, −5.606), (6.118, 1.799), (4.390, 2.577), and (−33.044, 0.258) for honey samples from the specific geographical origin (Figure 4).

The total correct classification rate was 97.7% using the original and 77.3% using the cross-validation method, both considered satisfactory classification results. The respective classification rates using the cross-validation method were: 71.4, 66.7%, 70.6%, 87.5%, and 100%, for citrus honey form Egypt, citrus honey from Morocco, citrus honey from Greece, citrus honey from Spain, and other nectar honeys from Greece, respectively. The overall error in each case was 28.6%, 33.3%, 29.4%, 12.5%, and 0%. Regarding the precision of the expanded LDA classification model, from the initial seven citrus honey samples from Egypt five were allocated correctly to Egypt, one to Morocco and one to Greece. In case of citrus honey samples from Morocco, four samples were allocated correctly to Morocco, one to Egypt and one to Greece. Regarding citrus honey samples from Greece, twelve were correctly allocated to Greece, three to Egypt, one to Morocco, and one to Spain. Concerning citrus honey samples from Spain, from the initial eight samples seven were allocated correctly to Spain and one to Greece. Finally, the additional nectar honey samples from Greece were all allocated correctly to Greece (Table 3). 

As is clearly shown, the additional honey samples introduced in the statistical analysis did not drastically affect the original classification model obtained for citrus honey from Egypt, Morocco, Greece, and Spain, as the new honey samples were classified in their initial group to 100%. Similarly, the volatile compounds that contributed most to the discrimination of citrus and nectar honey were lilac aldehyde D (absolute correlation value of 0.71), dill ether (absolute correlation value of 0.38), 2-methylbutanal (absolute correlation value of 0.34), cis-linalool oxide (absolute correlation value of 0.28), heptane (absolute correlation value of 0.23), 1-nonanol (absolute correlation value of 0.11), and ethyl heptanoate (absolute correlation value of 0.09).

## 4. Discussion

Considering citrus honey samples, the lilac isomers (I-IV) are of great importance, since these volatile compounds hold a high proportion in the total volatile fraction of citrus honey. It has been documented in previous studies [3,4,8] that these volatile compounds may potentially be considered as “key volatile compounds” in citrus honey. However, in more recent studies it was reported that lilac aldehydes could potentially contribute to the aroma of Spanish sunflower and thyme honey [6] and of Turkish pine honey [19]. Some other aldehydes such as octanal, nonanal, and decanal have been also identified in citrus honey [3,4,8]. In the present study, nonanal and decanal could aid to the geographical discrimination of citrus honey (Table 1). 2-Methylbutanal (which comprises a leucine- and isoleucine-derived volatile) was identified only in Egyptian citrus honey samples. Benzaldehyde, with an almond-like and pleasant aroma was identified in higher amount (mg/kg) in Moroccan and Greek citrus honey samples compared to Egyptian and Spanish citrus honey samples. However, the other nectar honey samples studied had the highest amount (mg/kg) of benzaldehyde. This compound has previously been reported to contribute to the volatile fraction of Greek and Spanish citrus honey [3,4,8].

Regarding furan derivatives, furfural (furan-2-carbaldehyde) and 5-methyl-2-furaldehyde are owed to the thermal pre-treatment of honey during the HS-SPME [15], heat processing or storage, and should not be considered as markers of botanical or geographical origin, as reported by Kadar et al. [4]. Even though significant differences were detected in the composition (mg/kg) of furfural, with respect to geographical origin of citrus honey, we are in line with the report of Kadar et al. [4], and this volatile compound was not considered as a marker of citrus honey geographical origin. 

Alpha-4-dimethyl-cyclohexene-1-acetaldehyde is a volatile compound that has been considered to contribute to the aroma of Spanish citrus honey [8]. Indeed, in the present study this volatile compound was identified in all citrus honey samples, and in significantly lower amount (mg/kg) in the other nectar honey samples.

Regarding esters, ethyl octanoate, ethyl nonanoate, and ethyl dodecanoate contribute to the geographical origin discrimination of citrus honey (Table 1). Methyl anthranilate (MA, 2-aminobenzoic acid methyl ester) was identified in all citrus honey samples in similar amounts (mg/kg), and therefore, could not be used as a potential indicator of geographical origin of citrus honey. MA is commonly used as a flavoring agent for its pleasant fruity odor. Its characteristic aroma can be described as fruity, concord grape, and musty, with a floral powdery nuance. This compound has been proposed as a fingerprint and an index of correct labelling of citrus honey [4,6]. Indeed, Escriche et al. [6] reported that MA was correlated with certain volatile compounds, such as 1-para-menthene-9-al, limonene, dill-ether, and ethyl linalool, and slightly lower with the four lilac aldehyde isomers. In our case, MA was significantly correlated (r = 0.31, *p* = 0.043 < 0.05) only with lilac aldehyde C during the Pearson’s correlation test, with some other potential correlations with volatile compounds such as lilac aldehyde D, α-4-dimethyl-cyclohexene-1-acetaldehyde dill ether, and ethyl dodecanoate.

Certain hydrocarbons such as heptane, octane, and nonane, have previously been identified in European unifloral honey samples [3,4,5,19,20,21]. Among these volatile compounds, heptane and octane could contribute to the geographical discrimination of citrus honey, since these were identified in diverse amounts (mg/kg) (with respect, of course, to the geographical origin of citrus honey) (Table 1). It should be noted that the other nectar honey samples studied contained higher amounts (mg/kg) of octane. Escriche et al. [21] reported a higher amount (mg/kg) of octane for Spanish lemon blossom honey compared to orange blossom honey. Several alkanes are common in the cuticle of honeybees, so the presence in honey could be due to an animal origin/activity and not exclusively from plants. Indeed, Patrignani et al. [11] reported that aliphatic fatty acids probably originate from beeswax, and this could explain the presence of these compounds in honey. 

Dill ether ((3S,3aS,7aR)-3,6-dimethyl-2,3,3a,4,5,7a-hexahydrobenzofuran) has previously been identified in Greek citrus honey [3,5]. Dill ether was determined in a higher amount (mg/kg) in Moroccan citrus honey than in the other types (Table 1). 

As for ketones, 6-methyl-5-hepten-2-one and β-damascenone ((E)-1-(2,6,6-Trimethyl-1-cyclohexa-1,3-dienyl)but-2-en-1-one) were identified only in citrus honey from Greece. 6-Methyl-5-hepen-2-one has previously been identified as a volatile component in Spanish lemon and orange honey [21] in conformity with the results of the present study. β-damascenone was also identified in the other nectar honey samples. It is worth mentioning that β-damascenone was not identified in a recent study concerning the volatile profile of Spanish citrus, sunflower, and thyme honey [6], nor in a previous study concerning acacia, sunflower, and tilia honeys from Spain, Romania, and the Czech Republic [22].

Phenolic volatile compounds (benzeneethanol and benzeneacetonitrile) were only identified in studied nectar honey samples from countries other than Greece. Regarding 3,4,5-trimethylphenol, similar amount (mg/kg) was recorded for citrus and other nectar honey samples only from Greece. Escriche et al. [6] reported a similar isomer (2,4,5-trimethylphenol) to contribute to the aroma of Spanish citrus and thyme honey. 

Seven terpenes, 2,6,6-trimethyl-bicyclo [3.1.1]hept-2-ene (α-pinene), 1-methyl-4-(1-methyl-ethyl) benzene (para-cymene), dl-limonene ((4*R*)-1-methyl-4-prop-1-en-2-ylcyclohexene), herboxide (isomer II) (5-isopropenyl-2-methyl-2-vinyltetrahydrofuran), hotrienol ((E)-3,7-dimethylocta-1,5,7-trien-3-ol), cis-linalool oxide (2-[(2S,5R)-5-ethenyl-5-methyloxolan-2-yl]propan-2-yl carbamates), and linalool (3,7-dimethylocta-1,6-dien-3-ol), were also identified in diverse amounts (mg/kg) (Table 1), in conformity with previous studies in the literature concerning Greek and Spanish citrus honey [3,6,7,20]. Terpenoids comprise the major components of essential oils of the flowers and fruits of the *Citrus* genus [4] and the presence of these volatile compounds in citrus honey is associated with the honeybee feeding on the nectar of these plant materials.

Driving the hypothesis further, the potential of volatile compounds in combination with chemometrics for the geographical and botanical origin discrimination of honey is really very satisfactory. Previous studies in the literature are in accordance with the results and the hypothesis presented in the present study. More specifically, Castro-Vázquez et al. [8] differentiated Spanish citrus, rosemary, lavender, thyme, eucalyptus, and heather honey using inalool derivatives, limonyl alcohol, sinensal isomers, and α,4-dimethyl-3-cyclohexene-1-acetaldehyde, acetoin, 5-hydroxy-2,7-dimethyl-4-octanone, para-cymene derivatives, 3-caren-2-ol, spathulenol, hexanal, nerolidol oxide, coumarin, hexanol, benzene, and phenolic compounds in combination with PCA. The first four components (PC1-PC4) explained 71% of the total variance. Patrignani et al. [11] reported that 3,8-para-menthatriene; cyclopropylidenemethylbenzene; 1,1,6-trimethyl-1,2-dihydronaphthalene; 1,2,4-trimethylbenzene; α-pinene; isopropyl 2-methylbutanoate; cymene; 2,6-dimethyl-1,6-octadiene; 3-methyloctane; 1-(1,4-dimethyl-3-cyclohexen-1-yl)ethanone; terpinolene; ethyl 2-phenylacetate; naphthalene; and seven unknown compounds could be used to classify Argentinean honeys according to their geographical origin with a prediction success of 96% using principal component analysis (PCA), redundancy analysis (RDA), and linear discriminant analysis (LDA). Duru et al. [19] reported that the application of PCA and hierarchical cluster analysis, as a comparative multivariate technique, on the volatile composition of Turkish pine honey showed that nonanal, nonanol, octanol, decanal, phenylacetaldehyde, benzaldehyde, octanal, α-pinene, 4-oxoisophorone, methyl salicylate, isopropyl myristate, limonene, and β-damascenone could be used as marker compounds of these type of honey. The first two components of PCA (PC1 and PC2) explained 41.3% of the total variance. Escriche et al. [6] applied PCA on the volatile composition of Spanish citrus, sunflower, and thyme honey, and reported that methyl anthranilate, 1-para-menthene-9-al, limonene, dill-ether, and ethyl linalool could be used for the differentiation of honeys. The total variance explained by the first two components (PC1 and PC2) was 67%. However, there is a lack in the relevant literature for a conclusive index originating from volatile compound composition to be used for discrimination purposes (geographical or botanical origin determination).

## 5. A New Index for the Geographical Origin Discrimination of Citrus Honey Based on the Ratio of Semi-Quantitative Data of Specific Volatile Compounds

The volatile compounds (variables) that contributed dynamically to the discrimination of citrus honey according to geographical origin were those with the higher absolute correlation during LDA, as LDA is a more efficient chemometric technique for discrimination purposes compared to PCA [16,17,18]. Therefore, the volatile compounds selected using LDA were used for the development of some specific ratios related majorly to citrus honey (R_ch_):

R_ch_= specific volatile compound semi-quantitative content/total volatile compounds semi-quantitative content (TSQVC) (Karabagias-Nayik index). (2)

The ratios were (i) lilac aldehyde D/TSQVC, dill ether/TSQVC, (ii) 2-methylbutanal/TSQVC, (iii) heptane/TSQVC, (iv) benzaldehyde/TSQVC, (v) α,4-dimethyl-3-cyclohexene-1-acetaldehyde/TSQVC, and (vi) herboxide (isomer II)/TSQVC with respect to the geographical origin of citrus honey. The ratio showed significant differences (*p* < 0.001) in relation to citrus honey geographical origin. Therefore, these ratios are proposed as complementary markers for citrus honey authentication, giving additional information on the richness of citrus honey aroma. In principle, these ratios could be used for the authentication of citrus or other honey types. Indeed, the flower and thyme honey samples studied (other nectar honey samples) had a significantly lower R value than citrus honey samples from other countries (R_nh_<<<R_ch_) (Table 1).

## 6. Conclusions

The findings of the current study are associated with the potential of volatile compounds (volatilome) to be used as original markers of citrus or nectar honey authentication in combination with machine learning algorithms. Even though semi-quantitative data of these volatile compounds were subjected to multivariate analysis, the obtained results can be considered satisfactory. In fact, the methodology applied provides a rapid analysis of some potential markers of citrus honey related to geographical origin in combination with machine learning algorithms. These statistically significant volatile compounds may potentially be used for the development of a new index of citrus honey geographical origin by producing specific ratios related to their semi-quantitative data. This for example, might be a rapid test of analysis. In this case, for the first time in the literature, we developed the Karabagias–Nayik ratio, indicative for citrus honey (Rch) of different geographical origin. This ratio may be generally used and for other honey types leading to new metrics for honey authentication.

## Figures and Tables

**Table 1:** Volatile compounds (mg/kg) identified in citrus and nectar honey of different geographical origin.

Volatile Compounds	RT (min)	RI	Citrus Honey from Egypt Avg (±SD)	Citrus Honey from Morocco Avg (±SD)	Citrus Honey from Greece Avg (±SD)	Citrus Honey from Spain Avg (±SD)	Nectar Honey from Greece Avg (±SD)	F
*Alcohols*								
1-Octanol	18.92	1069	nd	nd	nd	nd	0.003 (0.005)	4.21 **
1-Nonanol	21.04	1170	nd	nd	nd	nd	0.01 (0.01)	24.27 ***
***Aldehydes***								
2-Methylbutanal	6.79	<800	0.07 (0.09)	nd	nd	nd	nd	5.11 **
Furfural	13.25	837	0.13 (0.17)	0.14 (0.22)	0.01(0.01)	0.004 (0.01)	0.02 (0.02)	3.22 *
Benzaldehyde	16.75	978	nd	0.04 (0.05)	0.04(0.02)	0.004 (0.01)	0.07 (0.09)	3.65 *
Octanal	17.45	1005	nd	0.01 (0.02)	0.02(0.03)	nd	0.01 (0.01)	1.58 ns
Benzeneacetaldehyde	18.58	1059	nd	0.10 (0.09)	0.10(0.11)	0.003 (0.01)	0.46 (0.50)	5.88 **
Nonanal	19.64	1107	nd	0.09 (0.05)	0.15(0.11)	0.03 (0.06)	nd	7.58 ***
Lilac aldehyde (isomer I, A)	20.57	1147	0.13 (0.23)	0.06 (0.09)	0.05(0.14)	0.10 (0.09)	nd	0.89 ns
Lilac aldehyde (isomer II, B)	20.63	1149	0.06 (0.08)	0.06 (0.11)	0.24(0.22)	0.06 (0.06)	0.04 (0.06)	3.89 **
Lilac aldehyde (isomer III, C)	20.76	1156	0.14 (0.07)	0.27 (0.09)	0.31(0.35)	0.09 (0.08)	nd	2.75 *
Lilac aldehyde (isomer IV, D)	21.17	1176	nd	nd	nd	0.06 (0.02)	nd	49.19 ***
Decanal	21.77	1209	nd	nd	0.09(0.13)	nd	0.03 (0.02)	3.48 *
α,4-Dimethyl-3 cyclohexene-1-acetaldehyde	22.31	1234	0.05 (0.07)	0.07 (0.04)	0.003(0.01)	0.06 (0.04)	0.003(0.005)	6.41 ***
***Hydrocarbons***								
Heptane	9.49	<800	0.09 (0.05)	0.05 (0.06)	nd	0.04 (0.06)	nd	7.92 ***
Octane	12.26	800	0.01 (0.02)	0.01 (0.02)	0.03(0.02)	0.01 (0.01)	0.04 (0.02)	3.18 *
Nonane	14.94	900	nd	nd	0.02(0.03)	nd	0.001 (0.01)	1.56 ns
Delta-3-carene	17.94	1024	nd	nd	nd	nd	0.007 (0.005)	16.84 ***
Undecane	19.59	1099	nd	nd	nd	nd	0.007 (0.005)	16.84 ***
***Ethers***								
Dill ether	21.72	1203	0.11 (0.08)	0.13 (0.05)	nd	0.07 (0.03)	0.003 (0.005)	22.42 ***
***Esters***								
Ethyl hexanoate	17.25	993	nd	nd	nd	nd	0.003 (0.005)	4.21 **
Ethyl malonate	18.67	1044	nd	nd	nd	nd	0.003 (0.005)	4.21 **
Ethyl heptanoate	19.44	1092	nd	nd	nd	nd	0.007 (0.005)	16.84 ***
Ethyl octanoate	21.36	1191	nd	0.01 (0.01)	0.03 (0.02)	0.003 (0.009)	0.03 (0.02)	7.94 ***
Ethyl nonanoate	23.26	1290	0.03 (0.05)	0.01 (0.02)	0.04 (0.03)	0.02 (0.02)	0.07 (0.05)	2.89 *
Methyl anthranilate	24.74	1366	0.03 (0.05)	nd	0.02 (0.03)	nd	nd	2.06 ns
Ethyl decanoate	25.05	1389	0.04 (0.09)	nd	0.02 (0.02)	0.09 (0.02)	0.04 (0.01)	1.02 ns
Ethyl dodecanoate	28.42	1588	nd	nd	0.003 (0.004)	nd	0.01 (0.00)	12.42 ***
Ethyl hexadecanoate	34.94	1990	nd	nd	0.002 (0.004)	nd	nd	1.29 ns
***Ketones***								
6-Methyl-5-hepten-2-one	17.06	986	nd	nd	0.001 (0.002)	nd	nd	2.85 *
β-Damascenone	25.37	1401	nd	nd	0.003 (0.06)	nd	0.003 (0.005)	1.75 ns
***Phenolic compounds***								
Benzeneethanol	20.20	1129	nd	nd	nd	nd	0.07 (0.09)	7.16 ***
Benzeneacetonitrile	20.71	1154	nd	nd	nd	nd	0.04 (0.05)	7.35 ***
3,4,5-Trimethylphenol	24.10	1330	nd	nd	0.003 (0.005)	nd	0.002 (0.004)	1.16 ns
***Terpenes***								
α-Pinene	16.18	949	nd	nd	0.01 (0.09)	nd	0.01 (0.004)	3.38 *
Herboxide isomer II	17.55	1007	0.05 (0.05)	0.10 (0.13)	0.01 (0.01)	0.08 (0.06)	0.003 (0.01)	4.92 **
Para-cymene	18.13	1038	nd	0.03 (0.05)	0.03 (0.03)	0.03 (0.05)	nd	2.24 ns
dL-Limonene	18.25	1044	0.01 (0.02)	0.01 (0.01)	0.002 (0.003)	nd	nd	0.86 ns
cis-Linalool oxide	19.11	1077	0.08 (0.08)	0.11 (0.04)	0.02 (0.01)	0.04 (0.02)	0.003 (0.01)	9.02 ***
Linalool	19.54	1103	0.04 (0.05)	0.02 (0.02)	0.07 (0.03)	0.06 (0.02)	0.02 (0.03)	4.29 **
Hotrienol	19.63	1104	nd	0.01 (0.02)	nd	0.02 (0.03)	0.03 (0.08)	1.47 ns
**TSQVC**			1.07	1.33	1.33	0.87	1.05	
**R_ch_, R_nh_ (Karabagias-Nayik index)**			0.35a	0.29b	0.04c	0.27d	0.08e	

RT: retention time, RI: experimental retention indices values, nd: not determined, F: Value of the F-distribution, ns: not significant, * *p* < 0.05, ** *p* < 0.01, *** *p* < 0.001, Avg(±SD): average ± standard deviation values (mg/kg), TSQVC: total semi-quantitative volatile content (mg/kg), Rch: ratio of citrus honey, Rnh: ratio of nectar honey. Different letters in each row indicate statistically significant differences (*p* < 0.001) as indicated by one-sample *t*-test. α: alpha, β: beta.

**Table 2:** Cumulative variability explained by the 7 volatile compounds (factor variables, PCs) with eigenvalues greater than 1 during PCA.

Principal Component Analysis	Ethyl Dodecanoate (F1)	Lilac Aldehyde C (F2)	Alpha-4-Dimethyl-3-Cyclohexene-1-Acetaldehyde (F3)	Lilac Aldehyde D (F4)	2-Methylbutanal (F5)	Methyl Anthranilate (F6)	Para-Cymene (F7)
Eigenvalue	10.889	5.374	2.348	1.976	1.602	1.298	1.225
Variability (%)	34.029	16.795	7.339	6.174	5.006	4.056	3.829
Cumulative %	34.029	50.825	58.164	64.338	69.344	73.400	77.230

PCs: Principal components. The compound with the highest loading value is highlighted in the corresponding principal component.

**Table 3:** Citrus and nectar honey classification with respect to geographical origin using the significant (*p* < 0.05) volatile compounds and LDA.

LDA	Prediction Rate	Geographical Origin	Predicted Group Membership					Total Citrus and Nectar Honey Samples
Method			Citrus Honey from Egypt	Citrus Honey from Morocco	Citrus Honey from Greece	Citrus Honey from Spain	Other Nectar Honeys from Greece	
Original ^a^	Count	Citrus honey from Egypt	7	0	0	0	0	7
		Citrus honey from Morocco	0	6	0	0	0	6
		Citrus honey from Greece	0	0	17	0	0	17
		Citrus honey from Spain	0	0	1	7	0	8
		Other nectar honeys from Greece	0	0	0	0	6	6
	%	Citrus honey from Egypt	100.0	0.0	0.0	0.0	0.0	100.0
		Citrus honey from Morocco	0.0	100.0	0.0	0.0	0.0	100.0
		Citrus honey from Greece	0.0	0.0	100.0	0.0	0.0	100.0
		Citrus honey from Spain	0.0	0.0	12.5	87.5	0.0	100.0
		Other nectar honeys from Greece	0.0	0.0	0.0	0.0	100.0	100.0
Cross-validated ^b,c^	Count	Citrus honey from Egypt	5	1	1	0	0	7
		Citrus honey from Morocco	1	4	1	0	0	6
		Citrus honey from Greece	3	1	12	1	0	17
		Citrus honey from Spain	0	0	1	7	0	8
		Other nectar honeys from Greece	0	0	0	0	6	6
	%	Citrus honey from Egypt	71.4	14.3	14.3	0.0	0.0	100.0
		Citrus honey from Morocco	16.7	66.7	16.7	0.0	0.0	100.0
		Citrus honey from Greece	17.6	5.9	70.6	5.9	0.0	100.0
		Citrus honey from Spain	0.0	0.0	12.5	87.5	0.0	100.0
		Other nectar honeys from Greece	0.0	0.0	0.0	0.0	100.0	100.0

^a^ 97.7% of original method grouped cases correctly classified. ^b^ Cross-validation is performed only for those cases in the analysis. In the cross-validation method, each case is classified by the functions derived from all cases other than that particular case. ^c^ 77.3% of cross-validation method grouped cases correctly classified.

## Data Availability

Not applicable.

## Supplementary Materials

The following supporting information can be downloaded at: https://www.mdpi.com/article/10.3390/foods12030509/s1, Section I: Melissopalynological analysis: botanical origin of honey samples, Section II: Discrimination of citrus honeys according to geographical origin based on volatile compounds and machine learning algorithms, Table S1: Classification of citrus honey according to geographical origin using the statistically significant volatile compounds and LDA.
