# Supplementary material for: Machine Learning Algorithms Applied to Semi-Quantitative Data of the Volatilome of Citrus and Other Nectar Honeys with the Use of HS-SPME/GC–MS Analysis, Lead to a New Index of Geographical Origin Authentication
Source: Foods. 2023 Jan 22;12(3):509. doi: 10.3390/foods12030509 (PMC9914582; doi:10.3390/foods12030509)
Supplement: Supplementary file 1 [file foods-12-00509-s001.zip › foods-2118048-supplementary.pdf]

Supplementary Material for Online Review

Machine Learning Algorithms Applied to Semi-Quantitative Data of the Volatilome of Citrus and Other Nectar Honeys with the Use of HS-SPME/GC–MS Analysis Lead to a New Index of Geographical Origin Authentication

Section I

Melissopalynological analysis-Botanical origin of honey samples

- ✓ Melissopalynological analysis: Citrus honey samples from Egypt, Morocco, and Greece (Arta and Argos)
- ✓ Declaration in packaging: Citrus honey samples from Spain (El Brezal)
- ✓ Declaration by professional beekeepers: Nectar honey samples (flower and thyme honey) from Greece

Melissopalynological analysis

Melissopalynological analysis

For qualitative melissopalynological analysis, 10 g of each honey sample was diluted in 20 ml of distilled water and centrifuged at 3000 rpm for 10 min. The sediment of the solution was dried at 40 °C and mounted on Entellan Rapid (Merck, 1.07961.0500). The honeydew elements and pollen grains were counted and identified in 20 optical areas at 200× magnification using an OLYMPUS BX 40 light microscope. The determination of the botanical origin was based on the relative frequencies of nectariferous species (Von der Ohe et al., 2004; Karabournioti et al., 2007). Pollen types from nectarless species were recorded and counted separately. Only the pollen grain types with frequencies higher than 1% were considered.

Melissopalynological analysis results

❖ Citrus honey samples from Egypt (N=7)

1.
Umbelliferae 36%
Citrus spp.28%
Eucalyptus spp.22%
Trifolium alexandrinum 8%
Pheonix sp.3%
<1% *Casuaria* sp., *Ephorbia* sp.
Nectarless: *Gramineae*

2.
Citrus sp.36%
Umbelliferae 36%
Eucalyptus spp.24%
Trifolium alexandrinum 1%
Pheonix spp.1%
<1% Compositae, *Brassica* sp., *Diplotaxis* sp.
Nectarless: *Gramineae*

3.
Umbelliferae 29%
Citrus spp.18%
Trifolium alexandrinum 18%
Eucalyptus sp.14%

Brassica spp.12%
Compositae 3%
<1% *Pheonix* spp.
Nectarless: *Zea mays*

4.
Citrus spp.38%
Umbelliferae 32%
Compositae 18%
Trifolium alexandrinum 6%
Brassica spp.3%
<1% *Sesamum* spp., *Diplotaxis* sp.
Nectarless: *Zea mays, Gramineae*

5.
Citrus spp.42%
Eucalyptus spp.28%
Umbelliferae 12%
Compositae 8%
Trifolium alexandrinum 3%
Brassica spp.1%
Pheonix spp.1%
Nectarless: *Zea mays, Gramineae*

6.
Eucalyptus spp.45%
Citrus spp.32%
Umbelliferae 8%
Compositae 6%
Trifolium alexandrinum 3%
Brassica spp.1%
Pheonix spp.1%
Nectarless: *Zea mays, Gramineae*

7.
Umbelliferae 33%
Citrus spp.32%
Eucalyptus sp.22%
Compositae 6%
Trifolium alexandrinum 1%
Brassica spp.1%
Pheonix spp.1%
Nectarless: *Zea mays, Gramineae*

- **Citrus honey samples from Morocco (N=6)**

1.
Trifollium spp. 32%
Boraginaceae 28%
Eucalyptus spp. 18%
Umbelliferae 12%
Citrus spp. 8%
<1% Compositae, *Euphorbia* sp., *Artemisia* sp.

Nectarless: *Olea europaea, Quercus* sp

2.
Eucalyptus spp. 28%
Trifollium spp. 22%
Boraginaceae 17%
Citrus spp. 12%
Eryngium spp.10%
Umbelliferae 3%
Compositae 3%
Brassica spp. 1%
Urginea spp.1%
<1% Rosaceae, *Peganum* spp.
Nectarless: *Olea europaea, Quercus* spp.

3
Boraginaceae 83%
Citrus spp. 8%
Compositae 3%
Brassica spp. 3%
Eryngium spp.1%
Umbelliferae 1%
<1% *Euphorbia* spp., *Eucalyptus* spp., *Vicia* spp., *Thymus* spp.
Nectarless: *Olea europaea*

4.
Boraginaceae 43%
Umbelliferae 30%
Compositae 13%
Citrus spp. 8%
Brassica spp. 2%
Trifollium spp. 2%
<1% *Peganum* spp., *Euphorbia* spp.
Nectarless: *Olea europaea*

5.
Eucalyptus spp. 28%
Trifollium spp. 22%
Boraginaceae 22%
Citrus spp. 12%
Umbelliferae 5%
Compositae 3%
Brassica spp. 3%
Lilliaceae 1%
<1% *Eucalyptus* spp., *Vicia* spp.
Nectarless: *Olea europaea, Quercus* spp.

6.
Eucalyptus spp. 23%
Citrus spp. 18%
Trifollium spp. 17%
Boraginaceae 16%
Umbelliferae 16%

Compositae 4%
Brassica spp. 3%
<1% *Urginea* sp., *Ephedra* spp.
Nectarless: *Olea europaea*

- Citrus honey samples from Greece (N=17)

Region of Arta (N=7)

1.

Erica spp.41%

Citrus spp. 22%

Castanea sativa 20%

Umbeliferae 6%

Phlomıs spp. 6%

Arbutus spp. 2%

Nectarless: *Querqus* spp.

2.

Trifollium spp. 75%

Citrus spp. 12%

Castanea sativa 12%

Nectarless: *Querqus* spp.

3.

Trifollium spp. 60%

Erica spp.12%

Citrus spp. 10%

Boraginaceae 7%

Compositae 6%

Nectarless: *Querqus* spp.

4.

Erica spp.40%

Citrus spp. 18%

Phlomıs spp. 16%

Brassica spp. 11%

Vicia spp. 5%

Rosaceae 5%

Umbeliferae 1%

Nectarless: *Olea europaea*, *Querqus* spp.

5.

Phlomis spp. 31%

Erica spp. 16%

Trifollium spp.16%

Citrus spp. 8%

Umbeliferae 6%

Thymus capitatus 5%

Compositae 5%

Brassica spp.4%

Rosaceae 4%

Nectarless: Olea europaea, *Cistus* spp.

6. *Trifollium* spp.32%

Erica spp. 21%

Phlomis spp. 12%

Citrus spp. 9%

Brassica spp.7%

Compositae 4%

Umbeliferae 4%

Rosaceae 4%

Boraginaceae 2%

Liliaceae 1%

Nectarless: Olea europaea, *Cistus* spp.

7. *Trifollium* spp. 58%

Erica spp.10%

Citrus spp. 12%

Boraginaceae 6%

Compositae 7%

Nectarless: *Querqus* spp.

❖ Region of Argos (N=10)

1. *Brassica* spp. 45,0%
Erica spp.24,5%
Lilliaceae12,5%
Compositae 7%
Oxalis spp. 7%
Citrus spp. 2%
Nectarless: *Olea europaea*

2. *Brassica* spp. 49,0%
Erica spp.20%
Lilliaceae10,5%
Compositae 6,5%
Phlomis spp. 6,0%
Citrus spp. 2,5,0%
Nectarless: *Olea europaea*

3. *Brassica* spp. 35%
Trifollium spp.32%
Erica spp.8%
Lilliaceae 6,5%
Compositae 4,5%
Phlomis spp. 4%
Citrus spp. 2%
Nectarless: *Olea europaea*

4. *Brassica* spp. 45%
Trifollium spp.17%
Erica spp.16%
Lilliaceae 6%
Phlomis spp. 2,5%
Citrus spp. 1,5%
Nectarless: *Olea europaea*

5. *Brassica* spp. 32%
Trifollium spp.30%
Lilliaceae 8%
Phlomis spp. 8%
Citrus spp. 5%
Oxalis spp. 4%
Compositae 4,0%
Boraginaceae 3,0%
Rosaceae 3,0%
Nectarless: *Olea europaea, Cistus* spp.

6. *Trifollium* spp.35%

Brassica spp. 2%

Lilliaceae 18%

Compositae 14%
Citrus spp. 5%
Erica spp. 2%
Boraginaceae 1%
Rosaceae 1%
Nectarless: *Olea europaea*

7. *Trifollium* spp.38%
Brassica spp. 31%
Compositae 19%
Citrus spp. 6%
Lilliaceae 2%
Boraginaceae 1,5%
Nectarless: *Olea europae*

8. *Brassica* spp. 40%
Compositae 24%
Trifollium spp.12%
Erica spp.10%
Citrus spp. 3%
Lilliaceae 2%
Oxalis spp. 2%
Boraginaceae 2%
Nectarless: *Olea europaea*

9. *Trifollium* spp.41%
Brassica spp. 22%
Compositae 8%
Lilliaceae 8%
Citrus spp. 5%
Oxalis spp. 4%
Boraginaceae 4%
Nectarless: *Olea europaea*

10. *Brassica* spp. 38%
Trifollium spp.29%
Compositae 14%
Boraginaceae 13%
Citrus spp. 3%
Nectarless: *Olea europaea.*

Section II

Discrimination of citrus honeys according to geographical origin based on volatile compounds and machine learning algorithms

MANOVA/LDA

MANOVA analysis was applied to the semi-quantitative data of volatile compounds of the 38 citrus honey samples to determine which volatile compounds are significant

for their geographical origin discrimination. Dependent variables included the 32 volatile compounds, while the geographical origin of honey samples (Egypt, Morocco, Greece, and Spain) was taken as the independent variable.

Pillai's Trace = 2.920 (F = 5.709, df = 96, $p<0.001$) (with observed power equal to 1.000), and Wilks' Lambda = 0.000 (F = 5.758, df = 96, *p<0001*) (with observed power equal to 0.995) index values showed the existence of a significant multivariable effect of the geographical origin of citrus honey on its volatile composition. Seventeen of the 32 volatile compounds were found to be significant ($p<0.05$) for the geographical origin discrimination of citrus honey. Afterwards, these 17 volatile compounds were subjected to LDA.

Results showed that three statistically significant discriminant functions were formed: Wilks' Lambda = 0.002, X^2 = 160.157, df = 51, $p<0.001$) for the first function; Wilks' Lambda = 0.035, X^2 = 88.585, df = 32, $p<0.001$) for the second; and Wilks' Lambda = 0.276, X^2 = 34.077, df = 15, $p<0.01$) for the third. A significant value of Wilks' Lambda index shows that the discriminant functions created are basic for the classification of the investigated groups of objects. In parallel, the multivariate effect of the geographical origin on the volatile composition of citrus honey samples is shown by the F-value tests.

The first discriminant function accounted for 59.5% of the total variance and had the highest eigenvalue (13.892) and canonical correlation (0.966). The second discriminant function had a significantly lower eigenvalue (6.822) and canonical correlation (0.934), while accounted for 29.2% of the total variance. Finally, the third discriminant function had the lowest eigenvalue (2.618) and canonical correlation (0.851) accounting for 11.2% of the total variance. All discriminant functions accounted for 100% of the total variance.

During LDA, the eigenvalue of the discriminant function is an essential parameter, since it provides information on how well the function differentiates the initial groups (geographical origin of citrus honey samples). What is also of great importance, are the group centroid values which comprise another essential parameter in LDA. The group centroid values are considered for the estimation of the classification ability of the LDA model and refer to the unstandardized canonical discriminant functions, evaluated at group means. The centroid values have two numbers which represent the coordinates (the abscissa is the first discriminant function and the ordinate is the second discriminant function) (Karabagias et al., 2017).

The group centroid values were: (0.046, -1.865), (3.190, -4.347), (-3.509, 0.778), and (5.024, 3.239), for citrus honey from Egypt, Morocco, Greece, and Spain (Figure 2).

The overall correct classification rate was 100% using the original and 78.9% the cross- validation method. The geographical origin classification rates using the cross-validation method were: 71.4, 50%, 88.2%, and 87.5% for citrus honey form Egypt, Morocco, Greece, and Spain, respectively. The individual classification rate for citrus honey from Morocco is considered poor.

From the initial 7 samples from Egypt, 5 were allocated correctly to Egypt, 1 in Morocco and 1 in Greece; In the case of citrus honey samples from Morocco, 3 samples were allocated correctly to Morocco, 2 to Egypt and 1 in Greece; The best classification results were obtained for citrus honey samples from Greece, where from the initial 17 samples, 15 were correctly allocated to Greece and 2 to Egypt; Finally, very good classification results were also obtained for the citrus honey samples from Spain, as from the initial 8 samples 7 were allocated correctly to Spain and 1 in Morocco (Table 2). During the LDA analysis the volatile compounds with the higher discrimination power were: lilac aldehyde D (absolute correlation value of 0.57), dill ether (absolute correlation value of 0.32), 2-methylbutanal (absolute correlation value

of 0.41), heptane (absolute correlation value of 0.37), benzaldehyde (absolute correlation value of 0.37), alpha-4-dimetyhyl-3-cyclohexene-1-acetaldehyde (absolute correlation value of 0.19), and herboxide second isomer (absolute correlation value of 0.17).

Table S1. Classification of citrus honey according to geographical origin using the statistically significant volatile compounds and LDA.

LDA Method	Prediction rate %	Geographical origin	Predicted Group Membership: Egypt	Morocco	Greece	Spain	Total citrus honey samples
Original[a]	Count	Egypt	7	0	0	0	7
		Morocco	0	6	0	0	6
		Greece	0	0	17	0	17
		Spain	0	0	0	8	8
	%	Egypt	100.0	0.0	0.0	0.0	100.0
		Morocco	0.0	100.0	0.0	0.0	100.0
		Greece	0.0	0.0	100.0	0.0	100.0
		Spain	0.0	0.0	0.0	100.0	100.0
Cross-validated[b,c]	Count	Egypt	5	1	1	0	7
		Morocco	2	3	0	1	6
		Greece	2	0	15	0	17
		Spain	0	1	0	7	8
	%	Egypt	71.4	14.3	14.3	0.0	100.0
		Morocco	33.3	50.0	0.0	16.7	100.0
		Greece	11.8	0.0	88.2	0.0	100.0
		Spain	0.0	12.5	0.0	87.5	100.0

[a]. 100.0% of original method grouped cases correctly classified. [b]. Cross-validation is done only for those cases in the analysis. In cross-validation method, each case is classified by the functions derived from all cases other than that particular case. [c].78.9% of cross-validated method grouped cases correctly classified.
